# Blood Clots Per Rectum in a Pediatric Patient With Salmonella Enteritis: A Case Report

**DOI:** 10.7759/cureus.27618

**Published:** 2022-08-02

**Authors:** Puja Shah, Arnikka Rubia, Nimrah Iqbal, Lisandra Gonzalez, Ratna B Basak

**Affiliations:** 1 Pediatrics, Brookdale University Hospital Medical Center, New York, USA; 2 Pediatrics, Maimonides Medical Center, New York, USA

**Keywords:** bloody stool, lower gi bleed, blood clots per rectum, hematochezia, salmonella enteritis

## Abstract

Infection is a common cause of hematochezia in children. While infectious diarrhea can present with bloody stools, it is rare to have the passage of frank blood clots per rectum in the pediatric population. This is a case of a seven-year-old male who presented with vomiting, diarrhea, severe abdominal pain, and passage of blood clots per rectum. As symptoms progressed, consideration of non-infectious causes was investigated and subsequently ruled out. The stool polymerase chain reaction (PCR) was positive for Salmonella species, while stool culture was negative for any enteropathogen. This report highlights the unusual occurrence of the passage of blood clots per rectum in a child with salmonella enterocolitis.

## Introduction

Acute gastroenteritis is a frequent cause of outpatient visits and hospitalizations in the USA, causing about 500,000 hospitalizations, and more than 5,000 deaths. While the most common cause of hematochezia in the pediatric population is infectious, a variety of other causes must be ruled out, including life-threatening etiologies [1].

Viruses are the most common etiology of diarrhea; however, it is the bacterial enteropathogens that result in bloody stools. The most common causes of infectious colitis causing hematochezia in children are Campylobacter and Salmonella. The differential diagnoses to consider for hematochezia are intussusception, inflammatory bowel disease, Meckel’s diverticulum, food protein-induced proctocolitis, antibiotic-associated colitis, and anal fissure [2]. Mesenteric ischemia can occur in patients with intussusception, incarcerated hernia, mesenteric thrombosis, or volvulus which can lead to hematochezia and are important surgical emergencies [3]. Toxic megacolon and hemolytic uremic syndrome are also potentially lethal causes of bloody stools.

We present a case of a seven-year-old child with vomiting, diarrhea, severe abdominal pain, with bloody diarrhea which progressed to the passage of frank blood clots per rectum. History and physical examination, supported by diagnostic investigations and stool polymerase chain reaction (PCR) positive for Salmonella, confirmed an infectious cause of hematochezia in the patient.

## Case presentation

A 7-year-old African American male was brought to the emergency department (ED) with complaints of watery diarrhea, intermittent crampy abdominal pain, and vomiting for four days. His physical examination was unremarkable. He was discharged after supportive treatment.

**Figure 1:**
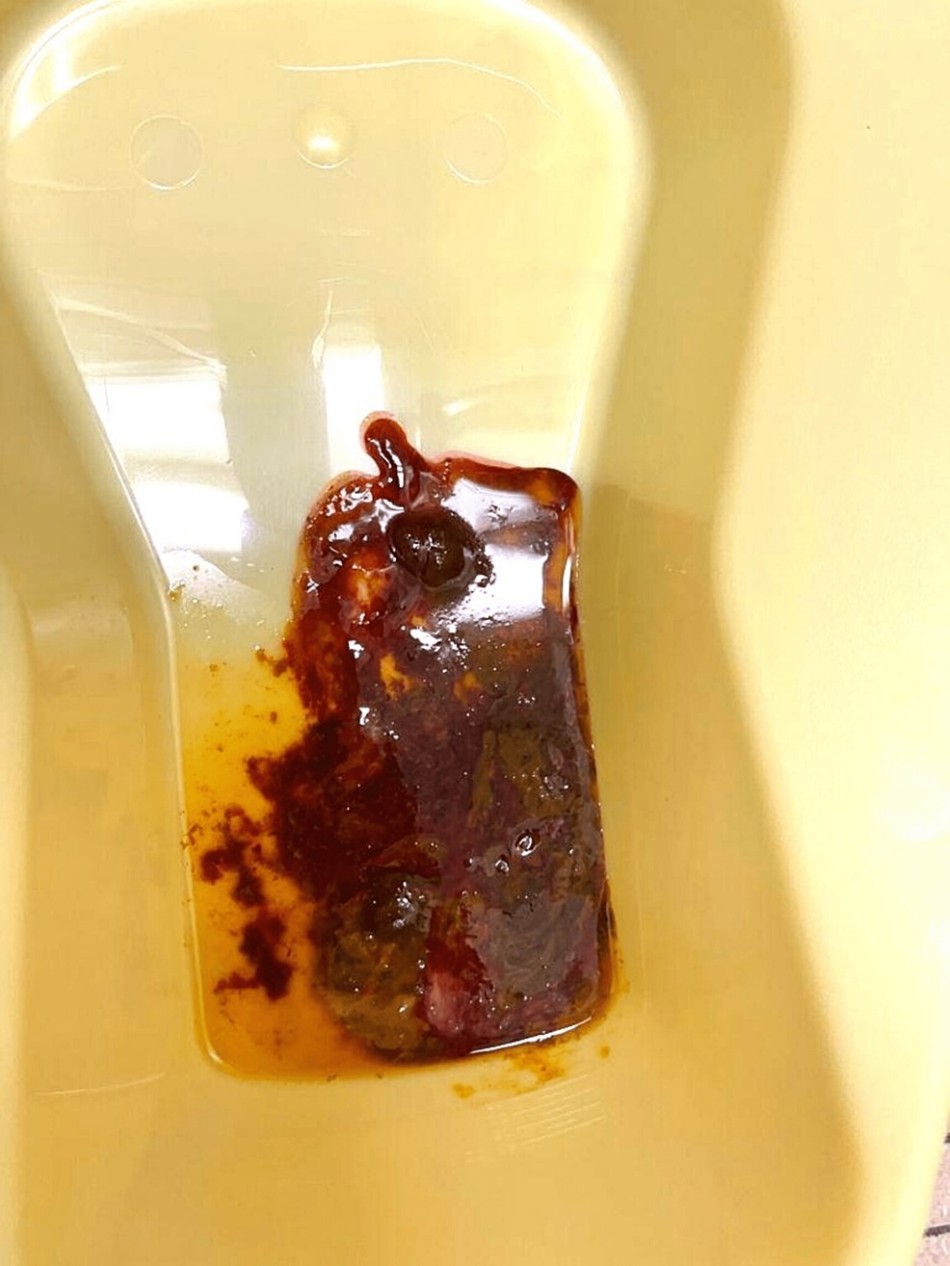
The image shows a stool mixed with blood.

He returned to the ED the following day with complaints of passing bloody stools (as shown in Figure 1) associated with left lower quadrant abdominal pain which was noted during defecation. There was no fever, joint pain, rashes, or rectal pain. There was no history of constipation, previous episodes of passing blood in stools, recent upper respiratory infection, antibiotic use, exposure to poultry, zoo animals, and sick contacts, or recent travels. His immunizations were up to date. He had no significant past medical or surgical history. His father and paternal aunt had a history of surgically corrected isolated malrotation. He had normal vital signs without signs of dehydration. He had localized left-lower quadrant abdominal tenderness and the rest of the physical exam was unremarkable.

Laboratory tests as in Table 1 showed normal hemoglobin (13.6 g/dl) and white cell count (6600 /μL), elevated lipase (854 U/L) and amylase (292 U/L), and slightly elevated C-reactive protein (CRP) (1.639 mg/dl) and erythrocyte sedimentation rate (ESR) (28 mm/hr). Renal and liver functions were normal. The abdominal radiograph was unremarkable. Abdominal ultrasound showed a reactive enlarged ovoid node in the right lower quadrant and thickened bowel loops and suggested that intussusception can be ruled out.

**Table 1:** Observed and reference laboratory values

Laboratory Tests	Observed Values	Reference Values
Hemoglobin	13.6 g/dl	11.5 - 15.5 g/dl
White blood cell count	6600 /μL	4500 - 13000 /μL
Lipase	854 U/L	8 - 69 U/L
Amylase	292 U/L	23 - 92 U/L
C-reactive protein (CRP)	1.639 mg/dl	0.0 - 0.9 mg/dl
Erythrocyte sedimentation rate (ESR)	28 mm/hr	0 - 15 mm/hr

**Figure 2:**
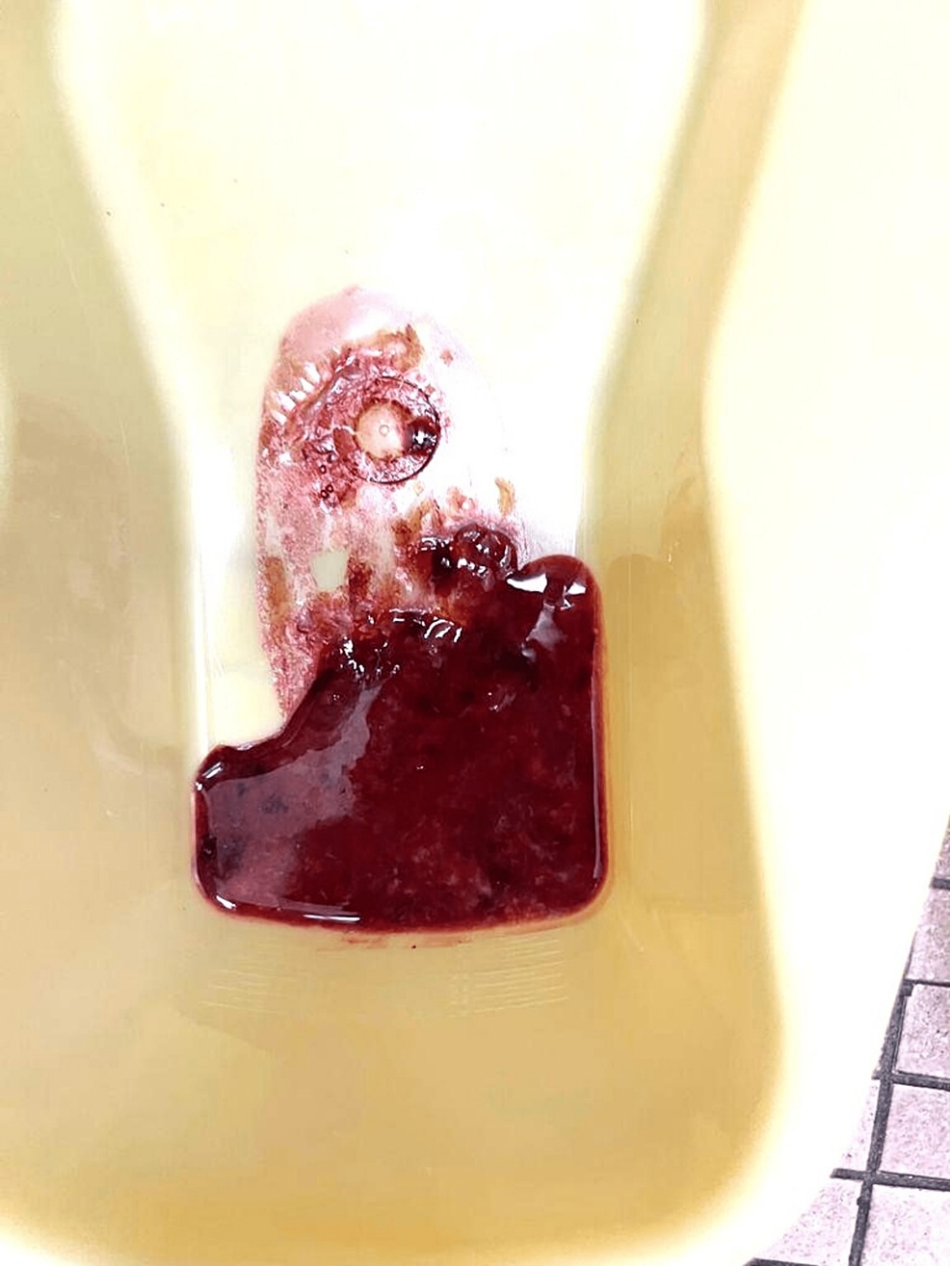
The image depicts the progression of hematochezia to bright red blood with visible blood clots.

After admission, the patient passed several blood clots per rectum as shown in Figure 2. The patient was kept nil per oral with maintenance intravenous fluids. Surgery and pediatric gastroenterology services were consulted. Possible differentials included infectious diarrhea, Meckel’s diverticulum, and intussusception.

The gastrointestinal panel PCR of the stool was positive for Salmonella species. However, stool culture was negative for Salmonella, Shigella, Campylobacter, Aeromonas, Plesiomonas, Vibrio, Escherichia coli 0157, and Yersinia. Fecal calprotectin was significantly elevated at 2,588 ug/gm (normal value: 0-120 ug/gm). Clostridium difficile antigen and toxin A and B were negative. There were no ova and parasites seen in the stool.

Meckel’s scan was negative for Meckel’s diverticulum. A repeat ultrasound of the abdomen three days after admission showed resolution of bowel wall thickening with no sonographic evidence of intussusception or pancreatitis. The patient’s stool frequency decreased, and he was discharged four days later on supportive management and gastroenterology follow-up. On a follow-up visit with gastroenterology, the patient’s bloody diarrhea was resolved. Upper gastrointestinal series was obtained outpatient due to concern for malrotation given family history, however, it was unremarkable. To date, the patient has had no recurrence of bloody stools.

## Discussion

Hematochezia is not commonly seen in the pediatric population in the western hemisphere and the etiology ranges from benign to life-threatening causes [2]. In children, the epidemiology is not well studied and only a few large series exist [2]. The most common causes of infectious diarrhea presenting with severe abdominal pain often with grossly bloody stools with minimal or no fever are infections with Shiga toxin-producing E. coli, Salmonella, Shigella, Campylobacter, and Yersinia enterocolitica [1]. One study revealed that while infectious colitis is the most common cause of hematochezia in children, Salmonella comes second to Campylobacter as the most common cause [2].

Both typhoidal Salmonella and non-typhoidal Salmonella (NTS) can be invasive. However, there is a paucity of data as to what particular subtypes of Salmonella are associated with risk for gastrointestinal bleeding [4]. The incidence of bleeding in typhoid colitis is 12.5% [5]. Risk factors for invasive nontyphoidal Salmonella infection are the following: infants from birth to three months of age and adults >50 years with a history of atherosclerosis, immunosuppressed, malnutrition, hemoglobinopathies, recent malaria, and cirrhosis [1]. Salmonella is transmitted via the fecal-oral route and once ingested, it can invade the intestinal epithelium and proliferate in the Peyer’s patches, prominently located in the terminal ileum [4, 5]. Ultrasonographic findings include thickened bowel loops and reactive lymphadenopathy. The terminal ileum is the most common site of bleeding, followed by the ileocecal junction, ascending colon, and transverse colon respectively [4].

Because hematochezia can be due to other life-threatening causes, non-infectious causes must be sought out. In the presented case, intussusception was ruled out due to the absence of signs of obstruction in the plain radiograph, and the absence of signs of intussusception on the sonogram. Ultrasound’s sensitivity and specificity for identifying intussusception are 97.9% and 97.8%, respectively [6]. Intussusception can be associated with a lead point such as Meckel diverticulum, polyp, nodular lymphoid hyperplasia, lymphoma, or bowel wall edema [3]. Meckel diverticulum is unlikely at this age and was ruled out with the negative Meckel’s scan.

Other fatal causes of hematochezia such as bowel ischemia leading to pneumatosis intestinalis, volvulus, incarcerated hernia, or mesenteric thrombosis must be considered. In our patient with a family history of malrotation, an upper gastrointestinal series was obtained which ruled it out. Hematochezia with abdominal pain can also be the presenting signs of Henoch-Schönlein purpura and these symptoms may precede the pathognomonic skin findings by up to one week [3]. Inflammatory Bowel Disease can also present with frank blood per rectum although it is usually associated with chronic symptoms such as weight loss, history of oral ulcers, anal fissures, chronic diarrhea, or constipation.

Patients presenting with fever or bloody diarrhea should be evaluated for pathogens for which antibiotics may confer a clinical benefit, particularly Salmonella enterica subspecies, Shigella, and Campylobacter [1]. However, identifying Salmonella as the etiology of infectious bloody diarrhea can be challenging due to various factors, such as the following: Firstly, the clinical features of salmonella enteritis are nonspecific and do not point to the etiology; secondly, routine microscopic stool examination is of limited utility as most children with salmonella enteritis have <5 polymorphonuclear cells per high power field [7]. Thirdly, there is insufficient data to support the diagnostic yield of fecal calprotectin measurement in patients with acute infectious diarrhea. Therefore, fecal leukocyte examination and stool lactoferrin detection should not be used to establish the cause of acute infectious diarrhea [1]. Another factor is that stool culture is currently considered to be the gold standard for the detection of enteric salmonella but despite having high specificity, it has low sensitivity (<50%) and is time-consuming [8]. On the other hand, stool PCR is highly sensitive, less dependent on the quality of the specimen, and a quicker method of detection but comes at the cost of lower specificity compared to stool culture [1,8].

Table 2 shows a comparison of characteristics of different PCR test methods and stool culture in detecting Salmonella in the stool. InvA and Salmonella tetrathionate respiration genes (ttr) in stool are the ones being detected by PCR. Multiplex PCR is a well-validated test to detect the above-mentioned genes, and a study was conducted that compared it to monoplex PCR, a test that includes a selenite broth pre-culture step for Salmonella before DNA extraction. All PCR tests have higher sensitivity than the stool culture for detecting Salmonella. Although the specificity of stool culture is almost 100%, all PCR tests also have high specificity and the results come back in a shorter period. However, PCR testing is more costly [8].

**Table 2:** Comparison of various characteristics between stool culture and different PCR tests. qPCR: real-time polymerase chain reaction; ttr: tetrathionate respiration gene; InvA: Salmonella invasion gene A

	Stool culture	Monoplex		Multiplex	
		-qPCR ttr	-qPCR InvA	-qPCR ttr	-qPCR InvA
Sensitivity	62.88%	99.53%	95.06%	90.30%	89.41%
Specificity	99.99%	95.45	90.31%	99.30%	98.00%
Result expected	3-7 days	1.5 days	1.5 days	0.5 days	0.5 days
Costing	Low	High	High	Very high	Very high

Our literature search has revealed several case reports of massive gastrointestinal bleed and hematochezia as a complication of invasive Salmonella typhi infections in adults and children [4-6]. The passage of blood clots in the setting of salmonella enterocolitis in the pediatric population is uncommon and epidemiologic studies are limited [2]. Our case highlights the importance of keeping salmonella enterocolitis in the differential diagnoses of children presenting with frank bleeding per rectum with the passage of blood clots, despite negative stool culture.

## Conclusions

Hematochezia in the pediatric population is uncommon and epidemiological studies are limited. Most cases of bloody diarrhea in the pediatric population do not require an extensive diagnostic work-up as the majority are self-resolving and can be managed conservatively. However, it is imperative to identify the etiology of those presenting with hematochezia as it could be life-threatening, and may require antimicrobial therapy, or urgent surgical management.

Although infectious diarrhea is the most common cause of hematochezia in children, salmonella infection presenting with the passage of blood clots per rectum is not common. Stool PCR to identify enteropathogens causing diarrhea is a highly sensitive rapid test and could replace stool culture.
